# Generalized Tetanus in a Canadian Farmer Following Orthopedic Surgery

**DOI:** 10.3390/idr14020033

**Published:** 2022-04-13

**Authors:** Utkarsh Chauhan, Anukul Ghimire, Milan Raval, Curtiss Boyington, Adrienne Haponiuk, Gregory Koller, Jeffrey Korzan, Elaine Yacyshyn

**Affiliations:** 1 Faculty of Medicine and Dentistry, University of Alberta, Edmonton, AB T6G 2R7, Canada; uchauhan@ualberta.ca; 2 Department of Medicine, University of Alberta, Edmonton, AB T6G 2G3, Canada; anukul@ualberta.ca; 3 Department of Medicine, Division of Infectious Diseases, Red Deer Regional Hospital, Red Deer, AB T4N 4E7, Canada; mraval@ualberta.ca; 4 Department of Medicine, Division of Infectious Diseases, University of Alberta, Edmonton, AB T6G 2G3, Canada; crb@ualberta.ca; 5 Department of Emergency Medicine, University of Alberta, Edmonton, AB T6G 2B7, Canada; ahaponiuk@ualberta.ca; 6 Department of Medicine, Division of Rheumatology, University of Alberta, Edmonton, AB T6G 2G3, Canada; gkoller@ualberta.ca; 7 Department of Radiology & Diagnostic Imaging, University of Alberta, Edmonton, AB T6G 2B7, Canada; jkorzan@ualberta.ca

**Keywords:** *Clostridium tetani*, tetanus, trismus, vaccination, immunoglobulin, risk factors

## Abstract

Tetanus is extremely rare in developed countries. We report the first documented case of tetanus in the province of Alberta since 2016: a farmer that developed trismus, shoulder stiffness, and fevers eight days following orthopedic surgery. Tetanus immunoglobulin elicited rapid recovery. We highlight risk factors, pathogenesis, epidemiology, and diagnostic challenges.

## 1. Case Report

A 66-year-old man presented to a rural emergency department in Alberta, Canada with throat pain and shoulder stiffness. Twelve days before presentation, he underwent an uncomplicated total left hip replacement for advanced primary osteoarthritis. Eight days later, he developed drenching night sweats, subjective fevers with chills, and a sore throat. The next day, he experienced painful muscle spasms and stiffness in his upper extremities: first in his hands, then progressing very rapidly to the shoulders, neck, and jaw. Upon recording a fever of 38.1 °C, the patient was transferred to a tertiary care centre.

The patient’s medical history was notable for hypertension, gastroesophageal reflux disease, autoimmune gastritis, and gout. Medications included nifedipine, telmisartan, sildenafil, pantoprazole, naproxen for gout as needed, tramadol for post-operative pain, and enoxaparin injections for post-operative deep vein thrombus prophylaxis. The patient owned farms and tended to elk, cattle, bison, and seed crops. He consistently wore gloves and denied any recent injuries. He had not received any vaccinations or boosters for more than 15 years. He denied history of intravenous drug use. He denied any recent travel.

On examination, his temperature was 37.2 °C, heart rate was 78 beats per minute, respiratory rate was 16 breaths per minute, and oxygen saturation was 94% on room air. His blood pressure was 161/79 at the left arm and 138/79 at the right arm. The lungs were clear to auscultation. There was pain on palpation of the submandibular and anterior cervical chain but no lymphadenopathy. The patient was unable to fully open his jaw due to pain in the temporomandibular joints. Musculoskeletal exam revealed mild palpable shoulder effusion bilaterally. Strength of shoulder abduction was 2/5 bilaterally (limited by pain); strength of biceps, triceps, and wrist flexion and extension were 4/5 bilaterally. The left hip surgical site was pristine with no evidence of infection. Head to toe dermatologic examination did not reveal any other skin lesions. The remainder of the examination was unremarkable.

Laboratory studies showed a white cell count of 11,200/mm^3^ with 84% neutrophils. Hemoglobin was 10.4 g/dL. Serum sodium was 131 mmol/L. C-reactive protein was 220.5 mg/L (reference range, <8), and ferritin was 522 μg/L (reference range, 15 to 200). Creatine kinase and a liver panel were normal. Blood cultures were negative. Nucleic acid amplification tests for COVID-19, influenza, respiratory syncytial virus, parainfluenza, coronavirus, metapneumovirus, and adenovirus were negative.

**Figure 1:**
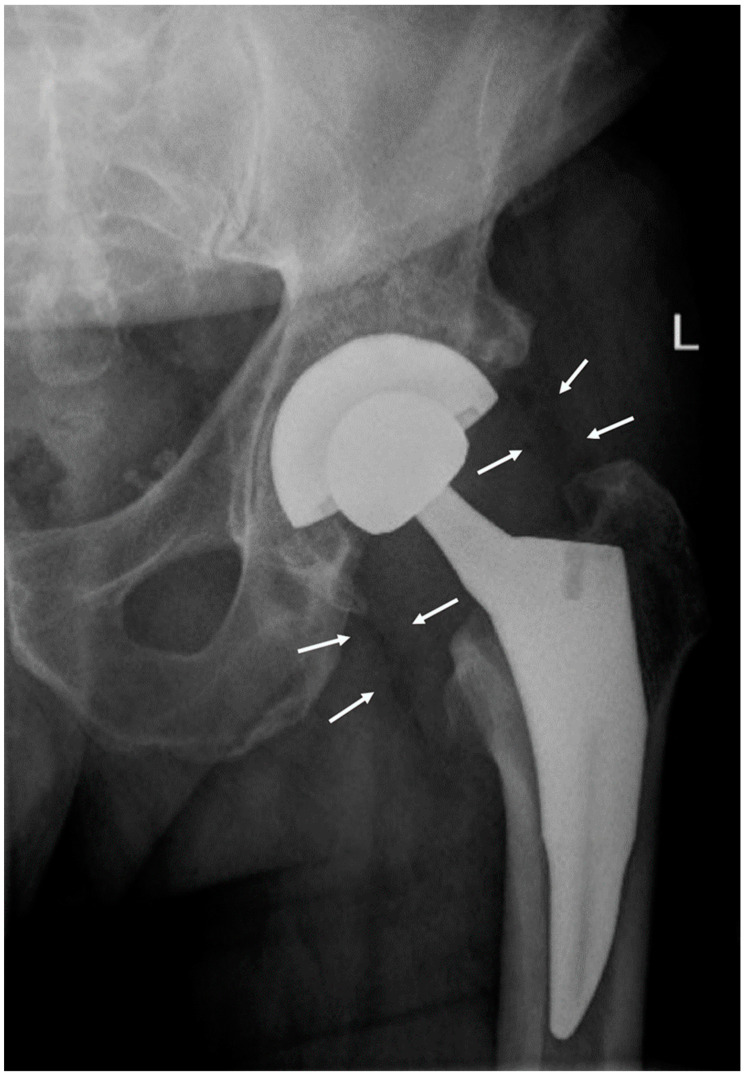
A hip radiograph demonstrating periprosthetic gas in soft tissue. Two foci of gas are outlined by white arrows; the largest focus of gas is at the lateral aspect of the hip. The patient underwent a total left hip replacement eight days prior to the onset of tetanus symptoms and demonstrated no other evidence of deep or superficial injury.

A radiograph of the hip showed periprosthetic gas in soft tissue (Figure 1). A CT scan of the neck and soft tissues along with an MRI of the C-spine were negative for compression or deep space abscess. Left shoulder arthrocentesis was negative for crystals with unremarkable chemistry. The blood pressure differential between arms prompted a CT angiogram of chest, abdomen, and pelvis that was negative for large vessel vasculitis.

The differential diagnosis included serotonin syndrome given the recent prescription of tramadol. However, the patient had only taken tramadol sparingly, and had no evidence of delirium. Neuroleptic malignant syndrome (NMS) was considered, but the patient was not confused and lacked suspect medications. A neurology consult excluded an unusual presentation of acute inflammatory demyelinating polyneuropathy (AIDP). The clinical presentation and normal creatine kinase did not fit with dermatomyositis, nor did the presentation fit with polymyalgia rheumatica (PMR).

The combination of trismus, increased muscle tone, fever, and elevated inflammatory markers suggested tetanus, which prompted an infectious disease consultation. Although the infectious disease team was unable to confirm the mechanism of infection, the constellation of symptoms, overdue vaccination, and evidence against competing hypotheses supported a working diagnosis of tetanus. Tetanus antitoxin levels were ordered to be drawn prior to the administration of tetanus immune globulin (TIG). His presumptive case was reported to Public Health. The patient was admitted and started on TIG and oral metronidazole. A CT of his left hip to evaluate for deep-seated infection or tissue necrosis was unremarkable. The patient was closely monitored with low threshold for ICU admission and benzodiazepines as needed for spasms.

Following administration of 3000 IU TIG, the patient showed marked improvement. He denied pain with palpation of his neck and shoulders. Tone in the neck and extremities returned to normal, as did strength (5/5) in the upper extremities.

By the third day following admission, the patient had received 6000 units of intramuscular TIG. He experienced a rapid and complete response. Unfortunately, tetanus antitoxin was drawn after administration of the initial dose, but titers demonstrated a low level of immunity (0.360 IU/mL, reference range, 0.1 to 0.5) despite TIG administration. He received a Tdap booster vaccination prior to discharge. As his prosthetic joint was considered a potential route of infection, a left hip ultrasound was conducted, but aspiration was unsuccessful. The patient was discharged on a six-week course of oral metronidazole, given the concern of possible hardware infection as the source of tetanus. He remained well at a six-month follow-up appointment with his infectious disease physician.

## 2. Discussion

We present the first case of tetanus, a notifiable disease, in the province of Alberta since 2016 [1]. The patient had known risk factors: agricultural exposure, a recent invasive procedure, and the absence of booster vaccination. Full dermatologic examination was negative for evidence of a wound that could be cultured. The clinical diagnosis was made robust by therapeutic trial: a profound and complete response to tetanus immunoglobulin.

Tetanus is an acute neurologic disease characterized by generalized rigidity and convulsive muscle spasms [2]. It is caused by an exotoxin, tetanospasmin, produced by the bacterium *Clostridium tetani*. *C. tetani* is spore-forming and anaerobic; its spores are widely distributed in soil and the intestines of both farm and domesticated animals, and it can be found on the skin of adults in agricultural areas [3].

*C. tetani* exposure often occurs through a cut or puncture wound by a contaminated object, a mechanism that delivers endospores to a low-oxygen environment [4]. Tetanus toxin is internalized at presynaptic membranes of motor neurons and undergoes retrograde transport to the central nervous system where it specifically blocks the release of GABA and glycine from inhibitory neurons. This leads to uncontrolled firing of motor neurons and the typical manifestations of tetanus. The usual incubation period is eight days, but can range from one day to several months [5].

Generalized tetanus accounts for approximately 80% of cases [6,7] of tetanus. The first sign is typically painful trismus (“lockjaw”), and usually follows a descending pattern with neck stiffness, odynophagia, and rigid pectoral, abdominal, and calf muscles. Historic descriptions include opisthotonos and “risus sardonicus”, a fixed smile with elevated eyebrows from facial muscle contraction [8]. Patients also present with fever, high blood pressure, and episodic tachycardia [2]. Muscle spasms occur frequently.

Complications of tetanus include laryngospasms that may interfere with breathing, fractures from sustained contractions, and autonomic instability that may cause arrhythmias [2]. Even with modern treatment, 11% of tetanus cases are fatal. Aspiration pneumonia is a common finding on autopsy, though 20% of tetanus deaths present no obvious pathology.

As isolating *C. tetani* from a wound is only possible in 30% of tetanus cases, the diagnosis is entirely clinical [2,6]. Modern techniques include PCR testing for tetanospasmin, but this test is costly with limited accessibility [9].

Vaccination programs worldwide are responsible for the declining incidence and mortality from tetanus. Tetanus toxoid-containing vaccines (TTCV) are included in routine immunization programs worldwide, and are frequently administered during antenatal care [3]. Teenagers and adults are recommended to receive a tetanus booster every 10 years [10].

Tetanus is rare in developed countries. Canadian data from 1920–1940 report 26–55 annual deaths due to tetanus [10]. TTCV programs introduced in the 1940′s led to a steep decline in morbidity and mortality. Between 2000 and 2013, an average of three cases have been reported annually, with only six deaths since 2000. The present case is the tenth reported incident in Alberta since the year 2000 for a population above 3 million [1]. Worldwide, tetanus accounts for approximately 50,000 deaths annually, with incidence dominated by agricultural regions in underdeveloped countries where contact with animal excreta is more common, along with inadequate immunization [8]. In rural areas of tropical countries within Asia, Africa, and South America, neonatal tetanus dominates, and tetanus mortality can exceed 50%.

This case presents a challenge to physicians, as tetanus is a disease seldom seen in developed countries due to the efficacy of vaccination programs. In our patient, a clinical diagnosis of tetanus required suspicion based on demographic and historical risk factors, rigorous elimination of rheumatologic, neurologic, and alternative infectious disease manifestations, and a rapid response to therapeutic trial. The mechanism for infection was unclear but may have been related to his recent total left hip replacement. Though this is considered a clean surgery, the classic incubation period of eight days fits well. Recent literature is scant for tetanus following insertion of prosthetic material, but post-operative tetanus following orthopedic [11] and gastrointestinal surgery using prosthesis [12,13] has been reported. Alternative explanations include unsterile enoxaparin injections and previous puncture injuries that had healed upon presentation.

Although tetanus is extremely rare in Canada, it is a life-threatening and treatable disease, and clinicians must be aware of risk factors for tetanus to recognize its atypical manifestations. Notably, older adults in rural areas often have limited access to primary care and routine vaccination. This demographic combines low levels of immunity with agricultural exposure characteristic of tetanus infection. Beyond diagnostic suspicion, we must continue a global effort to reduce the burden and mortality of tetanus through vaccination.

## 3. Conclusions

Tetanus is a rare disease entity among developed nations. However, the high mortality associated with this disease necessitates clinician awareness of typical risk factors and clinical features to initiate timely treatment. In our clinical scenario, a patient with agricultural exposure, a recent invasive surgical procedure, and lack of tetanus booster vaccination presented with trismus, progressive muscular spasms and stiffness. A rigorous evaluation involving rheumatologic, neurologic, and infectious disease specialists was conducted to exclude other potential diagnoses. The clinical diagnosis of tetanus was ultimately confirmed through a rapid and complete clinical response to therapeutic treatment trial.

## Data Availability

All available data is included in the manuscript.
